# Efficient Sensor Scheduling Strategy Based on Spatio-Temporal Scope Information Model

**DOI:** 10.3390/s23125437

**Published:** 2023-06-08

**Authors:** Yang Liu, Chen Dong, Xiaoqi Qin, Xiaodong Xu

**Affiliations:** State Key Laboratory of Networking and Switching Technology, Beijing University of Posts and Telecommunications, Beijing 100876, China; liu_young@bupt.edu.cn (Y.L.); xiaoqiqin@bupt.edu.cn (X.Q.); xuxiaodong@bupt.edu.cn (X.X.)

**Keywords:** internet of things, spatio-temporal scope information model, spatio-temporal correlation, sensor scheduling

## Abstract

In this paper, based on the information entropy and spatio-temporal correlation of sensing nodes in the Internet of Things (IoT), a Spatio-temporal Scope Information Model (SSIM) is proposed to quantify the scope of the valuable information of sensor data. Specifically, the valuable information of sensor data decays with space and time, which can be used to guide the system to make efficient sensor activation scheduling decisions for regional sensing accuracy. A simple sensing and monitoring system with three sensor nodes is investigated in this paper, and a single-step scheduling decision mechanism is proposed for the optimization problem of maximizing valuable information acquisition and efficient sensor activation scheduling in the sensed region. Regarding the above mechanism, the scheduling results and approximate numerical bounds on the node layout between different scheduling results are obtained through theoretical analyses, which are consistent with simulation. In addition, a long-term decision mechanism is also proposed for the aforementioned optimization issues, where the scheduling results with different node layouts are derived by modeling as a Markov decision process and utilizing the Q-learning algorithm. Concerning the above two mechanisms, the performance of both is verified by conducting experiments using the relative humidity dataset; furthermore, the differences in performance and limitations of the model are discussed and summarized.

## 1. Introduction

The Internet of Things connects the physical world and transforms physical objects from being traditional to smart [1]. There are a large number of sensing nodes in the IoT, whose role is to collect data and provide information to the upper layers to make relevant adjustments and decisions. Typical application scenarios include temperature and air quality monitoring in urban cities [2], smart traffic [3] and smart agriculture [4], etc. However, due to the cost constraints, most of the sensor nodes are usually low-cost sensors powered by batteries, which are very sensitive to power consumption, including micropower nodes and passive nodes. It has been one of the leading research problems to improve nodes’ sensing efficiency and prolong nodes’ working lives in these applications.

For certain applications that require a high degree of data accuracy, it is crucial to ensure the reliability of data, because significant data errors may lead to serious consequences, including large economic losses, etc. Therefore, some scholars have conducted research on improving the reliability of sensing data. Specifically, a novel unified Bayesian framework is proposed in [5] to enable the simultaneous estimation of a common parameter of interest and identification of multiple and possibly different types of anomalies. In addition, for some time-sensitive applications, the outdated data are not of much value to the system. Based on this idea, the concept of the Age of Information (AoI) [6] is proposed, which is defined as the time difference between the moment of the sensing data generation and acceptance by the receiver. On the one hand, the system expects nodes to send frequent updates so as to ensure the immediacy, but on the other hand, frequent updates can cause congestion in the system and consume too much energy of nodes. Therefore, numerous scholars have studied the trade-off strategy by combining AoI with various queuing and communication models. Specifically, the optimal service rates to minimize the average AoI in different queuing models are derived in [6]. The problem of minimizing the expected weighted sum AoI while simultaneously satisfying timely-throughput constraints is addressed in [7]. Reference [8] investigates the problem of minimizing the average and peak age of information (PAoI) under general interference constraints. A discrete-time queueing model to derive the exact distributions of the AoI and PAoI is proposed in [9].

The freshness of information is linearly decreasing in the definition of AoI, but there is a temporal correlation in some physical sources, which may cause the performance degradation due to information aging not being a linear function of time. Therefore, some scholars have proposed nonlinear functions about AoI and conducted related research. Reference [10] introduces a general age penalty function to characterize the level of dissatisfaction on data staleness. In [11], a general expression of the generating function of AoI and the PAoI metric is provided, which provides a methodology for analyzing general non-linear age functions. Exploiting the temporal correlation between consecutive samples of a Markov source, reference [12] considers a generalized incremental update scheme by sending differential updates.

In addition, the dense deployment of sensing nodes on the space inevitably causes the observations to be highly correlated in the spatial domain. Therefore, some scholars have studied the layout setting of nodes on the spatial domain and the spatial critical sampling rate, etc. The correlation between nodes is used to reconstruct the observed physical phenomena based on a fraction of all available sensor nodes in [13], where a framework for the analysis of sensor density is proposed. In [14], a theoretical analysis of spatio-temporal correlation characteristics of point and field sources is performed. Based on the confident information coverage (CIC) model, refs. [15,16] study the critical sensor density and find the optimal placement pattern to achieve complete coverage in randomly deployed networks. In addition, a node deployment scheme to maximize the network lifetime and ensure CIC with obstacles is proposed in [17].

Meanwhile, some scholars have simultaneously investigated the cooperative scheduling and spatio-temporal sampling rate among nodes in conjunction with the correlation of nodes in the time and space domain. More work related to this paper in this regard is described in detail in Section 1.2.

The main idea of this paper is to measure the value of the real-time useful information of sensor data in the temporal and spatial domains by using the spatio-temporal correlation of the sources. For example, the first data of sensors collected continuously in the time domain can bring the most amount of valuable information due to the lack of a priori knowledge, while the later data in the time domain can bring less valuable information than the first data due to the short time correlation of the physical properties of the sources, i.e., the first data can provide a certain amount of priori information for the later ones. Similarly, there is an overlap in the valuable information provided by the data acquired simultaneously by nodes close to each other in the spatial domain. In this paper, a model to measure the real-time valuable information of sensor data is established to represent the effective spatio-temporal scope of data information, and the optimal node scheduling strategy under different node layouts is investigated to improve the efficiency of sensing information.

### 1.1. Contributions and Paper Outline

Utilizing the spatio-temporal correlation of the sources, we establish an SSIM, which measures the valuable information of sensor data in the spatio-temporal domain. The main contributions are as follows.

1. Utilizing the spatio-temporal correlation of sensor nodes, a SSIM is proposed to quantify the valuable information of sensor data, which decays with space and time.
2. A single-step optimal decision-making mechanism is proposed. The possible scheduling results under different node layouts are analyzed, and a method to solve the boundary node distribution among various scheduling situations is provided.
3. A long-term optimal decision-making mechanism is proposed, which is modeled as a Markov decision process, and the Q-learning algorithm is utilized to solve the optimal scheduling results.
4. With a single-step mechanism, the approximate bounds for the node layout between partial scheduling results are obtained from the theoretical analysis and numerical calculation, which match with the simulation results. The optimal scheduling results with a long-term mechanism corresponding to different node layouts are obtained. Finally, the different performances of the two mechanisms are experimentally verified, and the advantages and limitations of each are summarized.

The rest of the paper is organized as follows: In Section 2, the system model and optimization problem are described. In Section 3, a single-step optimal mechanism is proposed for the established model, and the related theoretical analysis is made. In Section 4, a long-term optimal mechanism is proposed and the modeling and solution methods are described. In Section 5, numerical results and simulation results are presented. In Section 6, an experimental evaluation is performed. Finally, conclusions are drawn and discussions are made in Section 7.

### 1.2. Related Work

Among some recent studies, the following ones are more relevant to the work of this paper. Specifically, reference [18] considers a system consisting of two correlated information sources, and establishes a optimal time shift between the two sources’ updates. An energy-aware scheduling mechanism based on Deep Reinforcement Learning (DRL) is proposed in [19,20], which can prolong the lifetime of sensors. A measure for the freshness of information is proposed in [21], which uses the mutual information between the real-time source value and the delivered samples at the receiver. In [22,23], a mutual-information based Value of Information (VoI) framework is formalised to characterise how valuable the status updates are for Hidden Markov Models. An error-tolerable sensing (ETS) coverage, as the area where the estimated information is and with a smaller error than the target value, is defined in [24,25]. The performance of state updates is studied in [26,27], where the status is modeled as a time-varying Gauss–Markov Random Field (GMRF), and the estimation error is analyzed. In [28,29], an optimal scheduling policy over limited communication channels is derived that minimizes the time-average mean squared error (MSE). A novel timeliness metric with spatially and temporally correlative mutual information (STI) is proposed in [30], where an optimal update interval is found by solving an integer optimization problem. Assuming that the information can be commonly observed by multiple sensors, two multi-source information update problems are formulated in [31].

Table 1 shows in detail the comparison between our work and the above references. Although many scholars have studied the cooperative scheduling and sampling strategies among nodes by combining the spatio-temporal correlation of the sources, less attention has been devoted to all possible scheduling results and change rules of the scheduling results for different node layouts with an effective grasp of the whole region. It is the main motivation of this paper to study the possible optimal scheduling results for different node layout cases, and analyze the change law of optimal scheduling results for different node layouts.

## 2. System Model and Problem Formulation

### 2.1. System Model

**Figure 1:**
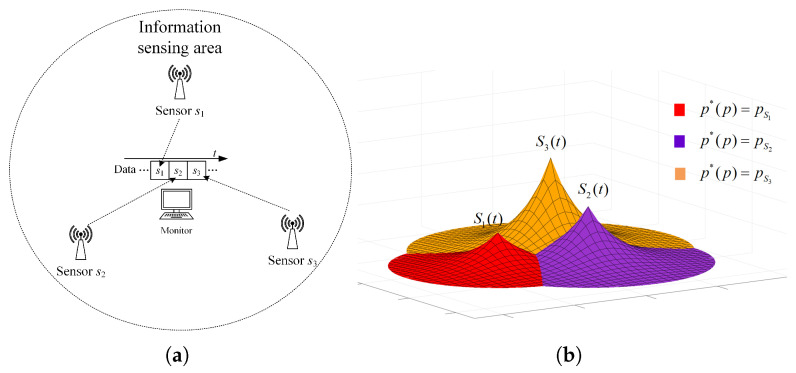
System model and spatio-temporal scope information map. (**a**) System model. (**b**) Scope information map.

In this paper, we mainly consider a simple sensing system composed of three sensing nodes, where the information sensing area is the whole two-dimensional (2D) plane, and the importance attached to each location in the area is the same, as shown in Figure 1a. For simplicity, it is assumed that the system periodically decides to activate a sensing node for information acquisition. The data transmission conditions are assumed to be ideal, ignoring the influence of data sending and transmission.

### 2.2. Spatio-Temporal Scope Information Model

In this paper, a Gaussian random field is assumed in the two-dimensional region to be measured, and the variables to be measured between any points in space conform to the joint Gaussian distribution. Without considering the node hardware acquisition error, for a certain node, let the random variable to be monitored corresponding to the spatial location of the node be *X*. The amount of valuable information that the first activation of the node can bring to *X* is the entropy h(X). Meanwhile, for any point *p*, let its corresponding random variable be $Y_{p}$. Denote the correlation coefficient as ρ, then the amount of information that the node data can provide at position *p* is

(1) $$I\left(p\right)=I\left(Y_{p};X\right)=h\left(Y_{p}\right)-h\left(Y_{p}|X\right)=-\frac{1}{2}\log\left(1-\rho^{2}\right).$$

Then, the total amount of information in the two-dimensional plane is

(2) $$I_{D}=\int_{0}^{2\pi }\int_{0}^{1}-\frac{1}{2}\log\left(1-\rho^{2}\right)\cdot \rho \cdot d\theta d\rho =-\frac{\pi }{2}\int_{0}^{1}\log\left(1-\rho^{2}\right)d\rho^{2}=\frac{\pi }{2}.$$

It can be observed from the above equation that although the information calculation result in Equation (1) tends to infinity when the correlation coefficient ρ tends to 1, the total scope information integral in the two-dimensional plane converges and can be calculated. In this paper, for simplicity, we consider the spatiotemporally separable covariance function [18] $\rho =e^{-\lambda_{d}\cdot d-\lambda_{t}\cdot t}$, where *d* represents the spatial distance, and *t* represents the time difference; in addition, $\lambda_{d}$ and $\lambda_{t}$ are the scaling parameters with respect to space and time, respectively. When the system has multiple nodes, there will be multiple nodes with spatio-temporal association at each location in the two-dimensional plane. In this paper, for the sake of simplicity, the system keeps only the latest sensor data of each node, and only the sensor data that can eliminate the most uncertainty and provide the most information is selected to provide a reference for a specific location. In other words, the amount of joint information provided by multiple nodes’ previous data together is not considered. That is, the amount of information available at any point *p* in the two-dimensional plane at moment *t* is

(3) $$I\left(p,t\right)=-\frac{1}{2}\log\left(1-e^{-2\lambda_{d}\cdot |p-p^{*}\left(p\right)|-2\lambda_{t}\cdot \left|t-t^{*}\left(p\right)\right|}\right),$$

where $p^{*}\left(p\right)$ is the node location coordinates of the most correlated sensor data for location *p*, and

(4) $$p^{*}\left(p\right)=\underset{p_{s_{i}}}{\arg\min}\lambda_{d}|p-p_{s_{i}}|+\lambda_{t}\left|t-t_{s_{i}}\right|,\;p_{s_{i}}\in S,$$

where *S* is the set of all node coordinates. Furthermore, $|t-t^{*}\left(p\right)|$ in Equation (3) is the value of AoI for the most correlated data at position *p*. According to the above equation, the spatio-temporal scope information map of the system at time *t* can be obtained as illustrated in Figure 1b, where the vertical height represents the amount of valuable information, and the location of the peak is the sensing node’s location.

At this moment, if the node $s_{i}$ is activated, the incremental information that can be obtained for each point *p* in the 2D plane is

(5) $$\begin{array}{cc} info_{gain}\left(p,s_{i}\right) & =h\left(Y_{p}|X_{past}^{*}\right)-h\left(Y_{p}|X_{s_{i}}\right)=\left[h\left(Y_{p}\right)-h\left(Y_{p}|X_{s_{i}}\right)\right]-\left[h\left(Y_{p}\right)-h\left(Y_{p}|X_{past}^{*}\right)\right]\\ & =-\frac{1}{2}\log\frac{1-e^{-2\lambda_{d}\cdot \left|p-p_{s_{i}}\;\;\;\right|\;\;\;}}{1-e^{-2\lambda_{d}\cdot \left|p-p^{*}\left(p\right)\right|-2\lambda_{t}\cdot AoI^{*}\left(p\right)}}, \end{array}$$

where $X_{s_{i}}$ is the random variable corresponding to position of node $s_{i}$, and $X_{past}^{*}$ is the random variable corresponding to the most spatiotemporally correlated node data at position *p*. In addition, $AoI^{*}\left(p\right)$ is the AoI value of the most correlated data. However, each time, a sensor node activated may acquire no new valuable information in certain regions. In other words, the previous data of other nodes provide more information in these regions than the node activated, and the result of the Equation (5) takes a negative value in these regions, which does not meet the definition of information. Because no more information can be provided, temporarily, no loss is caused.

Thus, the scope information increment in the whole two-dimensional plane of the activated node $s_{i}$ is

(6) $$\begin{array}{cc} \; & I_{gain}=\underset{D}{\;\int \int \;}\max\{info_{gain}\left(p,s_{i}\right),0\}d\sigma =\underset{D}{\;\int \int \;}\max\left\{-\frac{1}{2}\log\frac{1-e^{-2\lambda_{d}\cdot \left|p-p_{s_{i}}\;\;\;\right|\;\;\;}}{1-e^{-2\lambda_{d}\cdot \left|p-p^{*}\left(p\right)\right|-2\lambda_{t}\cdot AoI^{*}\left(p\right)}},0\right\}d\sigma . \end{array}$$

### 2.3. Problem Formulation

In this paper, it is the research objective to find the most efficient way of node activation scheduling given the node location layout and the spatio-temporal correlation coefficient of the region to be measured. That is, it is desired that the total amount of information mastered by the system for the entire two-dimensional plane has the maximum mean value in the time domain, as in the following equation

(7) $$\max\bar{I}=\lim_{T\to \infty }\;\frac{1}{T}\int_{0}^{T}[\underset{D}{\;\int \int \;}I(p,t)d\sigma ]dt.$$

Let the total amount of information held by the system at each activation node moment $t_{i}$ be $I_{i}$. In addition, let the total two-dimensional information decay function after the $i_{th}$ activation node of the system be $f_{i}\left(t\right)$, as follows

(8) $$f_{i}\left(t\right)=\frac{\underset{D}{\;\int \int \;}-\frac{1}{2}\log_{2}\left(1-e^{-2\lambda_{d}\cdot \left|p-p^{*}\left(p\right)\right|-2\lambda_{t}\cdot \left[AoI^{*}\left(p\right)+t\right]}\right)d\sigma }{\underset{D}{\;\int \int \;}-\frac{1}{2}\log_{2}\left(1-e^{-2\lambda_{d}\cdot \left|p-p^{*}\left(p\right)\right|-2\lambda_{t}\cdot AoI^{*}\left(p\right)}\right)d\sigma }.$$

Letting T=n∗Δt, the expression for the information mean can be further written as

(9) $$\begin{array}{c} \bar{I}=\lim_{n\to \infty }\;\frac{1}{n\cdot \Delta t}\sum_{i=1}^{n}I_{i}\cdot \int_{0}^{\Delta t}f_{i}\left(t\right)dt, \end{array}$$

and the following relationship holds for the total amount of two-dimensional information and the incremental information of the activated nodes

(10) $$I_{i+1}=I_{i}\cdot f_{i}\left(\Delta t\right)+I_{gain}\left(i\right),\;i=1,2,3,\cdots .$$

Recursive induction of the above equation leads to the following result

(11) $$\begin{array}{cc} I_{n} & =I_{gain}\left(n\right)+\sum_{i=1}^{n-1}\left[I_{gain}\left(i\right)\cdot \prod_{j=i}^{n-1}f_{j}\left(\Delta t\right)\right], \end{array}$$

(12) $$\begin{array}{c} \sum_{i=1}^{n}I_{i}\cdot \int_{0}^{\Delta t}f_{i}\left(t\right)dt=\sum_{i=1}^{n}\left\{I_{gain}\left(i\right)\cdot \left[\sum_{j=i}^{n}\left(\int_{0}^{\Delta t}f_{j}\left(t\right)dt\cdot \prod_{k=i}^{j-1}f_{k}\left(\Delta t\right)\right)\right]\right\}. \end{array}$$

The above equation is difficult to continue to derive, mainly because $f_{i}\left(t\right)$ is challenging to obtain a clear closed form; thus, it is proposed here to unify the total information decay of all moments approximately expressed as f(t), that is,

(13) $$f_{i}\left(t\right)\approx f\left(t\right),i=1,2,3\cdots .$$

The reason for such an approximate assumption here is mainly motivated by the consideration that the system tends to acquire more and smoother information, which leads to similar total information at each activation node moment; thus, causing the decay trend may be approximately the same. Then, Equation (12) can be further written as

(14) $$\int_{0}^{\Delta t}f(t)dt\cdot \sum_{i=1}^{n}\left(I_{gain}\left(i\right)\cdot \sum_{j=0}^{n-i}f{\left(\Delta t\right)}^{j}\right)=\frac{\int_{0}^{\Delta t}f(t)dt}{1-f(\Delta t)}\cdot \sum_{i=1}^{n}\left[I_{gain}\left(i\right)\cdot \left(1-f{\left(\Delta t\right)}^{n-i+1}\right)\right].$$

Since f(Δt)<1, for any positive number ε that is greater than 0 and small enough,

(15) $$\begin{array}{c} 1-f{\left(\Delta t\right)}^{n-i+1}<1-\varepsilon \Leftrightarrow i>n+1-\log_{f(\Delta t)}\varepsilon , \end{array}$$

thus, when *n* tends to infinity, the percentage of terms with $I_{gain}\left(i\right)$ coefficients less than 1−ε is

(16) $$\lim_{n\to \infty }\;\frac{\log_{f(\Delta t)}\varepsilon }{n}=0.$$

Therefore, the coefficients can be approximated by 1, the optimization problem becomes the following

(17) $$\begin{array}{c} \max\bar{I}=\frac{\int_{0}^{\Delta t}f(t)dt}{\Delta t\left(1-f(\Delta t)\right)}\cdot \lim_{n\to \infty }\cdot \frac{1}{n}\sum_{i=1}^{n}I_{gain}\left(i\right)\\ \;\;\;\;s.t.\;Se\left(t_{i}\right)=s_{i}\in \left\{s_{1},s_{2},s_{3}\right\},\;t_{i}=0,\Delta t,2\Delta t,\cdots ,n\Delta t, \end{array}$$

where $Se(t_{i})$ is the node activated at the moment $t_{i}$. From the above expression, the original problem is converted to the problem of maximizing the mean value of the incremental information.

## 3. Single-Step Optimal Mechanism

Regarding the above-mentioned problems, the first optimization mechanism that readily comes to mind is the single-step decision method, which means that the system computationally finds the sensing node that can obtain the most information increments at each discrete decision moment based on the known spatio-temporal information residual map, then makes it active and updates the spatio-temporal information map. The mathematical expression as a rule for each node activation $s_{i}$ can be written, as follows

(18) $$Se\left(t_{i}\right)=\underset{s_{i}\in \left\{s_{1},s_{2},s_{3}\right\}}{\arg\max}\;\underset{D}{\;\int \int \;}\max\{info_{gain}\left(p,s_{i}\right),0\}d\sigma .$$

According to whether the value of the latter term in the integral equation is greater than 0, the integration region can be divided into two parts, $D^{\prime }$ and $D^{\prime \prime }$; thus, the integral in Equation (18) is equivalent to

(19) $$\underset{D^{\prime }}{\;\int \int \;}-\frac{1}{2}\log\frac{1-e^{-2\lambda_{d}\cdot \left|p-p_{s_{i}}\;\;\;\right|\;\;\;}}{1-e^{-2\lambda_{d}\cdot \left|p-p^{*}\left(p\right)\right|-2\lambda_{t}\cdot AoI^{*}\left(p\right)}}d\sigma ,$$

where *D*${}^{\prime }$ and *D*${}^{\prime \prime }$ are the sets consisting of points that respectively satisfy the following conditions:

(20) $$\left\{\begin{array}{c} p\in D^{\prime },\;|p-p_{s_{i}}|-|p-p^{*}\left(p\right)|<\frac{\lambda_{t}}{\lambda_{d}}\cdot \left|t-t^{*}\left(p\right)\right|\\ p\in D^{\prime \prime },\;|p-p_{s_{i}}|-|p-p^{*}\left(p\right)|\geq \frac{\lambda_{t}}{\lambda_{d}}\cdot \left|t-t^{*}\left(p\right)\right|. \end{array}\right.$$

Furthermore, according to Equation (4), the following result can be obtained

(21) $$p^{*}\left(p\right)=p_{s_{k}},\;p\in S_{k},\;k=1,2,3.$$

where $S_{1}$, $S_{2}$ and $S_{3}$ are the sets of points that respectively meet the conditions as Equation (22),

(22) $$\left\{\begin{array}{cc} S_{1}=\left\{p\in D||p-p_{s_{1}}|-|p-p_{s_{2}}|<\frac{\lambda_{t}}{\lambda_{d}}\cdot \left({AoI}_{s_{2}}-{AoI}_{s_{1}}\right),\right. & \\ \left.|p-p_{s_{1}}|-|p-p_{s_{3}}|<\frac{\lambda_{t}}{\lambda_{d}}\cdot \left({AoI}_{s_{3}}-{AoI}_{s_{1}}\right)\right\} & \\ S_{2}=\left\{p\in D||p-p_{s_{2}}|-|p-p_{s_{1}}|<\frac{\lambda_{t}}{\lambda_{d}}\cdot \left({AoI}_{s_{1}}-{AoI}_{s_{2}}\right),\right. & \\ \left.|p-p_{s_{2}}|-|p-p_{s_{3}}|<\frac{\lambda_{t}}{\lambda_{d}}\cdot \left({AoI}_{s_{3}}-{AoI}_{s_{2}}\right)\right\} & \\ S_{3}=\left\{p\in D||p-p_{s_{3}}|-|p-p_{s_{1}}|<\frac{\lambda_{t}}{\lambda_{d}}\cdot \left({AoI}_{s_{1}}-{AoI}_{s_{3}}\right),\right. & \\ \left.|p-p_{s_{3}}|-|p-p_{s_{2}}|<\frac{\lambda_{t}}{\lambda_{d}}\cdot \left({AoI}_{s_{2}}-{AoI}_{s_{3}}\right)\right\}, & \end{array}\right.$$

in which the ${AoI}_{s_{1}}$, ${AoI}_{s_{2}}$ and ${AoI}_{s_{3}}$ are the AoI of the latest sensing data of each node. The increment of information that can be obtained by the activated node $s_{i}$ from the above expression can be written as

(23) $$\begin{array}{c} I_{gain}=\sum_{j=1}^{3}\underset{S_{j}}{\;\int \int \;}\max\{info_{gain}\left(p,s_{i}\right),0\}d\sigma =\sum_{j=1}^{3}\underset{{S_{j}}^{\prime }}{\;\int \int \;}info_{gain}\left(p,s_{i}\right)d\sigma , \end{array}$$

where $S_{j}^{\prime }$ is the set of points that meet the condition as

(24) $$\left|p-p_{s_{i}}\right|-\left|p-p_{s_{j}}\right|<\frac{\lambda_{t}}{\lambda_{d}}\cdot {AoI}_{s_{j}},\;p\in S_{j},\;j\in \left\{1,2,3\right\}.$$

**Figure 2:**
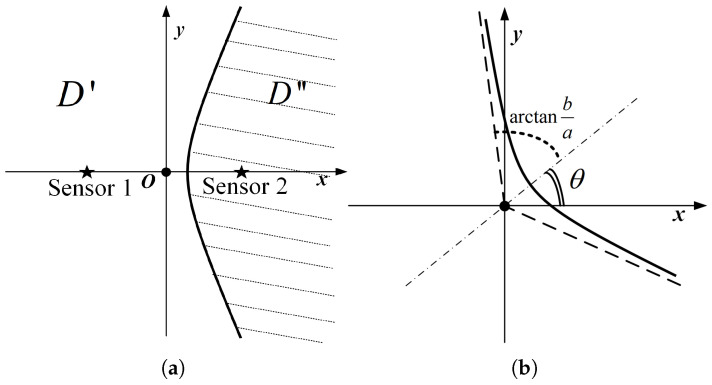
Schematic diagram of the distribution of set elements and boundary curves. (**a**) Schematic of the second activation node of the two-point system. (**b**) Schematic of the curve after rotating θ counterclockwise around the origin, where the dashed line represents the asymptote of the curve.

From the fact that the trajectory of a point with a constant distance difference to two points is a hyperbola, we can preliminarily determine that the boundary of the above set $D^{\prime }$ may contain several curves. For example, when the system only has two nodes, and $p_{s_{i}}$, $p^{*}\left(p\right)$ are taken as $p_{s_{1}}$, $p_{s_{2}}$, respectively, let the coordinates of node $s_{1}$ and node $s_{2}$ in 2D space be (−c,0), (c,0), as shown in Figure 2a. Abbreviating $\frac{\lambda_{t}}{\lambda_{d}}\cdot \Delta t$ to 2a, the inequality corresponding to the set $D^{\prime \prime }$ can be written as

(25) $$\begin{array}{cc} \; & 2c=d_{s_{1},s_{2}}>|p-p_{s_{1}}|-|p-p_{s_{2}}|\geq \frac{\lambda_{t}}{\lambda_{d}}\cdot \Delta t=2a>0\\ \Leftrightarrow & \frac{x^{2}}{a^{2}}-\frac{y^{2}}{c^{2}-a^{2}}\geq 1,\;x>0,\;c>a>0, \end{array}$$

and $D^{\prime \prime }$ is an empty set when c<a, i.e., $d_{s_{1},s_{2}}<\frac{\lambda_{t}}{\lambda_{d}}\cdot \Delta t$; in which case, a certain increment of information can be obtained in the whole 2D plane. However, it should be noted that activating node $s_{1}$ or node $s_{2}$ can obtain the same incremental information at this case, and there is no optimal activation scheme from the perspective of information acquisition only. Therefore, in the subsequent analytical modelling, the distance between the nodes must be at least greater than $\frac{\lambda_{t}}{\lambda_{d}}\cdot \Delta t$. The area distribution of $D^{\prime \prime }$ that can be obtained from Equation (25) is shown in the shaded part of Figure 2a. The remaining blank areas are where new information increments can be obtained, that is, the set $D^{\prime }$.

However, due to different node positions and different angles of the coordinate system, when the horizontal axis of the curve is not parallel to the x-axis, a 2D rotation matrix can be used to derive the equation of the curve $\frac{x^{2}}{a^{2}}-\frac{y^{2}}{b^{2}}=1$ after rotating θ counterclockwise around the origin as

(26) $$\left(\frac{\cos^{2}\theta }{a^{2}}-\frac{\sin^{2}\theta }{b^{2}}\right)x^{2}+\left(\frac{\sin^{2}\theta }{a^{2}}-\frac{\cos^{2}\theta }{b^{2}}\right)y^{2}+\sin2\theta \left(\frac{1}{a^{2}}+\frac{1}{b^{2}}\right)xy=1.$$

Moreover, the expression equation of a single curve may be conveniently written as only one of two forms y=f(x) or x=g(y), with different rotation angles. The primary method of discrimination is based on the maximum number of intersections of the two asymptotic lines of the curve with the horizontal and vertical lines. For example, as shown in Figure 2b, the curve can be expressed as x=g(y) when there is, at most, one intersection point between the two asymptotes of the curve and the line parallel to the x-axis. The applicability of the two expression forms can then be obtained as follows:

(27) $$\left\{\begin{array}{c} x=g\left(y\right)\Leftrightarrow \theta \in \left[0,\arctan\frac{b}{a}\right]\cup \left[\pi -\arctan\frac{b}{a},\pi \right]\\ y=f\left(x\right)\Leftrightarrow \theta \in \left[\frac{\pi }{2}-\arctan\frac{b}{a},\frac{\pi }{2}+\arctan\frac{b}{a}\right], \end{array}\right.$$

where $\frac{b}{a}$ is the absolute value of the slope of the hyperbola asymptote. It is easy to find that when $\arctan\frac{b}{a}<\frac{\pi }{4}$, i.e., $c<\sqrt{2}a$, the range of values of θ in Equation (27) does not completely cover [0,π]. In other words, there are certain ranges where a single curve cannot be expressed in the above two functional forms, and one solution in this case is to adjust the angle of the coordinate system for the subsequent expression.

**Figure 3:**
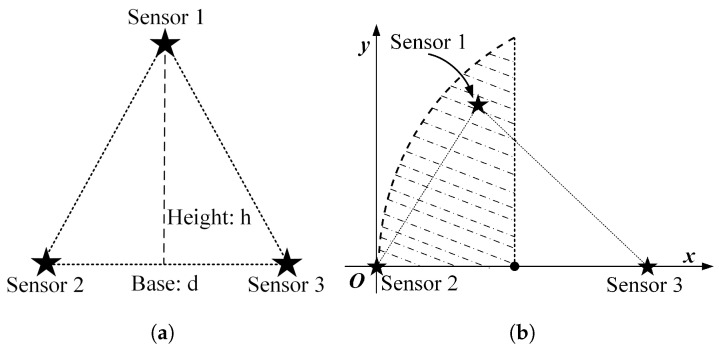
The location distribution of three nodes: (**a**) isosceles triangle; (**b**) general triangular.

When the three nodes present an equilateral triangular layout, it is easy to analyze that the optimal scheduling strategy under the single-step decision mechanism is an alternate activation of the three nodes in turn, according to the decay of information over time and the equal spatial correlation between individual nodes. However, it is essential to note that the above-mentioned alternate activation of nodes presupposes that the distance between nodes is greater than $\frac{\lambda_{t}}{\lambda_{d}}\cdot 2\Delta t$. The reason is that if the node distance is smaller than this value, the valuable information contained in the sensed data of each node can be covered by the data of the other nodes after two moments so that there exists the same increment of information available to two nodes at each decision moment. The following is a preliminary consideration to analyze the possible scheduling scenarios by changing the distance between only two nodes, keeping other conditions unchanged. The location distribution of the three nodes is assumed as shown in Figure 3a.

### 3.1. Three-Node Isosceles Triangle Layout

From the previous analysis, it is known that when the three nodes are laid out in an equilateral triangle, the node activation sequence can be set to 123123⋯. Thus from a long-term perspective, when node $s_{3}$ is activated, the AoI values of the latest sensing data of nodes $s_{1}$, $s_{2}$, $s_{3}$ are 2Δt, Δt, 3Δt, respectively, which are abbreviated as [213]. The amount of incremental information obtained by activating node $s_{3}$ is recorded as $info_{\left[2,1,3\right]}\left(s_{3}\right)$. Putting the AoI vector [213] into Equation (22), the formulas of the boundary curves for different regions in the information distribution map can be obtained, which satisfy the conditions as

(28) $$\left\{\begin{array}{c} C_{S_{1},S_{2}}:|p-p_{s_{2}}|-|p-p_{s_{1}}|=\frac{\lambda_{t}}{\lambda_{d}}\cdot \Delta t,\;d_{s_{1},s_{2}}>\frac{\lambda_{t}}{\lambda_{d}}\cdot \Delta t\\ C_{S_{1},S_{3}}:|p-p_{s_{1}}|-|p-p_{s_{3}}|=\frac{\lambda_{t}}{\lambda_{d}}\cdot \Delta t,\;d_{s_{1},s_{3}}>\frac{\lambda_{t}}{\lambda_{d}}\cdot \Delta t\\ C_{S_{2},S_{3}}:|p-p_{s_{2}}|-|p-p_{s_{3}}|=\frac{\lambda_{t}}{\lambda_{d}}\cdot 2\Delta t,\;d_{s_{2},s_{3}}>\frac{\lambda_{t}}{\lambda_{d}}\cdot 2\Delta t. \end{array}\right.$$

**Figure 4:**
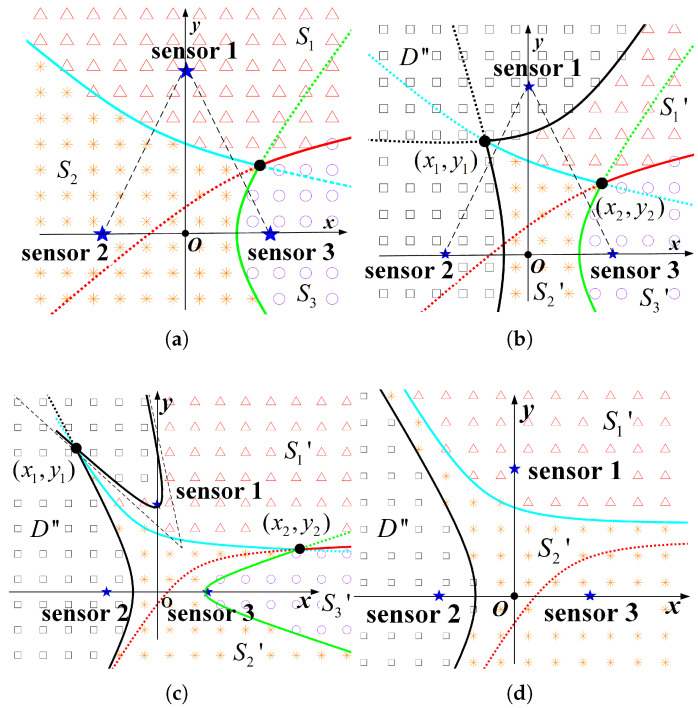
Spatio-temporal information distribution maps. The subscript indicates the AoI value of the current node data, and the brackets represent the currently active node. (**a**) $info_{\left[2,\;1,\;3\right]}$. (**b**) $info_{\left[2,\;1,\;3\right]}\left(s_{3}\right)$. (**c**) $info_{\left[2,\;1,\;3\right]}\left(s_{3}\right)$. (**d**) $info_{\left[2,\;1,\;3\right]}\left(s_{3}\right)$.

If one of the above conditions is not met, the corresponding boundary curve does not exist. Taking the midpoint of the base of the isosceles triangle as the origin and the base as the *x*-axis direction to establish a rectangular coordinate system, and setting the base length as *d* and the height of the triangle as *h*, the coordinates of the three nodes are (0,d), $\left(-\frac{d}{2},0\right)$, and $\left(\frac{d}{2},0\right)$, respectively. Then, the information residual distribution map of the system can be drawn according to Equations (22) and (28), as shown in Figure 4a, where the upper triangular, star and circular marker areas represent the sets $S_{1}^{\prime }$, $S_{2}^{\prime }$, and $S_{3}^{\prime }$; meanwhile, the solid lines are the boundaries of the different set regions. The three boundary curves necessarily intersect at a point, which can be proved by the geometric distance property of the hyperbola. When the rotation angle θ is 0 and the centre of the curve is at the origin, the general formula of a single curve is

(29) $$x=h\left(y,a,c,sign\right)=sign\cdot a\cdot \sqrt{1+\frac{y^{2}}{c^{2}-a^{2}}},$$

where sign is a symbolic variable that takes the value ±1, and *c* is the hyperbolic focal length. Thus, the set $S_{2}$ and $S_{3}$ boundary curve expression can be written as

(30) $$C_{S_{2},S_{3}}:x=h\left(y,2a,\frac{d}{2},1\right)=2a\cdot \sqrt{1+\frac{y^{2}}{\frac{d^{2}}{4}-4a^{2}}}.$$

The general expression of the $S_{1}$, $S_{2}$ boundary curve and the $S_{1}$, $S_{3}$ boundary curve can be written as Equation (31) by combining Equation (26) with Equation (27),

(31) $$\left\{\begin{array}{cc} y=f\left(x\right)=y_{o}-\frac{C}{2B}\cdot \left(x-x_{o}\right)\pm \frac{1}{\sqrt{\left|B\right|}}\sqrt{1+\left(\frac{C^{2}}{4B}-A\right){\left(x-x_{o}\right)}^{2}}, & \\ \theta \in [\frac{\pi }{2}-\arctan\frac{b}{a},\frac{\pi }{2}+\arctan\frac{b}{a}] & \\ x=g\left(y\right)=x_{o}-\frac{C}{2A}\cdot \left(y-y_{o}\right)\pm \frac{1}{\sqrt{\left|A\right|}}\sqrt{1+\left(\frac{C^{2}}{4A}-B\right){\left(y-y_{o}\right)}^{2}}, & \\ \theta \in \left[0,\arctan\frac{b}{a}\right]\cup \left[\pi -\arctan\frac{b}{a},\pi \right]. & \end{array}\right.$$

where $\left(x_{o},y_{o}\right)$ are the coordinates of the center point of the corresponding curve, and θ is the rotation angle of curve. In addition, *c* is the hyperbolic focal length, and

(32) $$\left\{\begin{array}{c} a=\frac{\lambda_{t}\cdot \Delta t}{2\lambda_{d}},\;b=\sqrt{c^{2}-a^{2}},\;C=\sin2\theta \left(\frac{1}{a^{2}}+\frac{1}{b^{2}}\right)\\ A=\frac{\cos^{2}\theta }{a^{2}}-\frac{\sin^{2}\theta }{b^{2}},\;B=\frac{\sin^{2}\theta }{a^{2}}-\frac{\cos^{2}\theta }{b^{2}}. \end{array}\right.$$

For the ease of subsequent expression, the Equation (31) is abbreviated to the following form:

(33) $$\left\{\begin{array}{c} y=f\left(x,a,c,x_{o},y_{o},\theta ,sign\right),\theta \in \Theta_{1}\\ x=g\left(y,a,c,x_{o},y_{o},\theta ,sign\right),\theta \in \Theta_{2}, \end{array}\right.$$

where sign takes the value ±1, representing the selection of positive or negative signs in the two expressions in Equation (31). Then, the $S_{1}$, $S_{2}$ and $S_{1}$, $S_{3}$ dividing lines can be obtained as Equations (34) and (35).

(34) $$C_{S_{1},S_{2}}:\left\{\begin{array}{c} y=f\left(x,a,\frac{\sqrt{h^{2}+d^{2}/4}}{2},\;-\frac{d}{4},\frac{h}{2},\arctan\left(\frac{2h}{d}\right),1\right),\\ \arctan\left(\frac{2h}{d}\right)\in \left[\frac{\pi }{2}-\arctan\frac{b}{a},\;\frac{\pi }{2}+\arctan\frac{b}{a}\right]\\ x=g\left(y,a,\frac{\sqrt{h^{2}+d^{2}/4}}{2},-\frac{d}{4},\frac{h}{2},\arctan\left(\frac{2h}{d}\right),1\right),\\ \arctan\left(\frac{2h}{d}\right)\in \left[0,\;\arctan\frac{b}{a}\right]\cup \left[\pi -\arctan\frac{b}{a},\;\pi \right]. \end{array}\right.$$

(35) $$C_{S_{1},S_{3}}:\left\{\begin{array}{c} y=f\left(x,a,\frac{\sqrt{h^{2}+d^{2}/4}}{2},\frac{d}{4},\frac{h}{2},\pi -\arctan\left(\frac{2h}{d}\right),-1\right),\\ \arctan\left(\frac{2h}{d}\right)\in \left[\frac{\pi }{2}-\arctan\frac{b}{a},\frac{\pi }{2}+\arctan\frac{b}{a}\right]\\ x=g\left(y,a,\frac{\sqrt{h^{2}+d^{2}/4}}{2},\frac{d}{4},\frac{h}{2},\;\pi -\arctan\left(\frac{2h}{d}\right),1\right),\\ \arctan\left(\frac{2h}{d}\right)\in \left[0,\arctan\frac{b}{a}\right]\cup \left[\pi -\arctan\frac{b}{a},\pi \right]. \end{array}\right.$$

The intersection of the three curves can be solved by combining the equations of the three curves, namely $C_{S_{1},S_{2}}$, $C_{S_{1},S_{3}}$, and $C_{S_{2},S_{3}}$. A quartic equation can be obtained by combining two of the curve formulas. Since the incremental information formula cannot calculate the exact analytical result, the numerical calculation can also be used to find the approximate numerical solution when solving intersections. The multiple solutions are then verified using another curve formula, and the solution that satisfies the condition is selected.

When node $s_{3}$ is activated, the conditions regarding the set $D^{\prime \prime }$ can be determined by the previous analysis method, as follows:

(36) $$\left\{\begin{array}{c} |p-p_{s_{3}}|-|p-p_{s_{1}}|\geq \frac{\lambda_{t}}{\lambda_{d}}\cdot AoI_{s_{1}}=\frac{\lambda_{t}}{\lambda_{d}}\cdot 2\Delta t\\ |p-p_{s_{3}}|-|p-p_{s_{2}}|\geq \frac{\lambda_{t}}{\lambda_{d}}\cdot AoI_{s_{2}}=\frac{\lambda_{t}}{\lambda_{d}}\cdot \Delta t. \end{array}\right.$$

Then, when the conditions $d_{s_{1},s_{3}}\geq \frac{\lambda_{t}}{\lambda_{d}}\cdot 2\Delta t$ and $d_{s_{2},s_{3}}\geq \frac{\lambda_{t}}{\lambda_{d}}\cdot 2\Delta t$ are met, two boundary curves of $D^{\prime \prime }$ can be obtained, as shown in Figure 4b, where the rectangular, upper triangular, star, and circular marker regions represent the sets $D^{\prime \prime }$, $S_{1}^{\prime }$, $S_{2}^{\prime }$, and $S_{3}^{\prime }$, respectively, and the set $S_{j}^{\prime }$ is defined, as shown in Equation (24). The coordinates of the intersection points of curves are set as $\left(x_{1},y_{1}\right)$, $\left(x_{2},y_{2}\right)$, respectively, as shown in the Figure 4b. Let the valuable incremental information that can be obtained at the position (x,y) be

(37) $$\begin{array}{c} info_{s_{e},s_{p}}\left(x,y\right)=-\frac{1}{2}\log\frac{1-e^{-{2\lambda }_{d}\cdot \sqrt{{\left(x-x_{s_{e}}\right)}^{2}+{\left(y-y_{s_{e}}\right)}^{2}}}}{1-e^{-{2\lambda }_{d}\cdot \sqrt{{\left(x-x_{s_{p}}\right)}^{2}+{\left(y-y_{s_{p}}\right)}^{2}}-{2\lambda }_{t}\cdot AoI_{s_{p}}}}, \end{array}$$

where $s_{e}$ is the currently active node, and $s_{p}$ is the node with information residuals at that location. Then, the valuable information increments that can be acquired in regions $S_{1}^{\prime }$, $S_{2}^{\prime }$ and $S_{3}^{\prime }$ are shown in Equations (38)–(40), respectively,

(38) $$\begin{array}{cc} \underset{S_{1}^{\prime }}{\;\int \int \;}info_{s_{3},s_{1}}\left(x,y\right)dxdy= & \int_{x_{1}}^{x_{2}}dx\int_{C_{S_{1}^{\prime },S_{2}^{\prime }}}^{C_{S_{1}^{\prime },D^{\prime \prime }}}info_{s_{3},s_{1}}\left(x,y\right)dy+\int_{x_{2}}^{\inf}dx\int_{C_{S_{1}^{\prime },S_{3}^{\prime }}}^{C_{S_{1}^{\prime },D^{\prime \prime }}}info_{s_{3},s_{1}}\left(x,y\right)dy, \end{array}$$

(39) $$\begin{array}{cc} \underset{S_{2}^{\prime }}{\;\int \int \;}info{}_{s_{3},s_{2}}\left(x,y\right)dxdy= & \int_{-\infty }^{y_{2}}dy\int_{C_{S_{2}^{\prime },D^{\prime \prime }}}^{C_{S_{2}^{\prime },S_{3}^{\prime }}}info{}_{s_{3},s_{2}}\left(x,y\right)dx+\int_{x_{1}}^{x_{2}}dx\int_{y_{2}}^{C_{S_{1}^{\prime },S_{2}^{\prime }}}info{}_{s_{3},s_{2}}\left(x,y\right)dy\\ - & \int_{y_{2}}^{y_{1}}dy\int_{x_{1}}^{C_{S_{2}^{\prime },D^{\prime \prime }}}info{}_{s_{3},s_{2}}\left(x,y\right)dx, \end{array}$$

(40) $$\begin{array}{cc} \underset{S_{3}^{\prime }}{\;\int \int \;}info{}_{s_{3},s_{3}}\left(x,y\right)dxdy= & \int_{-\infty }^{y_{2}}dy\int_{C_{S_{2}^{\prime },S_{3}^{\prime }}}^{+\infty }info{}_{s_{3},s_{3}}\left(x,y\right)dx+\int_{x_{2}}^{\inf}dx\int_{y_{2}}^{C_{S_{1}^{\prime },S_{3}^{\prime }}}info{}_{s_{3},s_{3}}\left(x,y\right)dy, \end{array}$$

where

(41) $$\left\{\begin{array}{c} C_{S_{1}^{\prime },S_{2}^{\prime }}:f\left(x,a,\frac{\sqrt{h^{2}+d^{2}/4}}{2},-\frac{d}{4},\frac{h}{2},\arctan\left(\frac{2h}{d}\right),1\right)\\ C_{S_{1}^{\prime },D^{\prime \prime }}:f\left(x,2a,\frac{\sqrt{h^{2}+d^{2}/4}}{2},\frac{d}{4},\frac{h}{2},\pi -\arctan\left(\frac{2h}{d}\right),1\right)\\ C_{S_{1}^{\prime },S_{3}^{\prime }}:f\left(x,a,\frac{\sqrt{h^{2}+d^{2}/4}}{2},\frac{d}{4},\frac{h}{2},\pi -\arctan\left(\frac{2h}{d}\right),-1\right)\\ C_{S_{2}^{\prime },S_{3}^{\prime }}:h\left(y,2a,\frac{d}{2},1\right),\;\;C_{S_{2}^{\prime },D^{\prime \prime }}:h\left(y,a,\frac{d}{2},-1\right). \end{array}\right.$$

However, it should be noted that the curve formulas in the above expressions, $C_{S_{1}^{\prime },S_{2}^{\prime }}$, $C_{S_{1}^{\prime },S_{3}^{\prime }}$, and $C_{S_{1}^{\prime },D^{\prime \prime }}$ all take the first expression form y=f(x), which is applicable to most of the node layout cases, but not to all. In addition, there are a few special layouts where a curve cannot be uniquely expressed as either of the two forms y=f(x) and x=g(y). For example, when $\frac{\sqrt{h^{2}+d^{2}/4}}{2}<\sqrt{2}\cdot 2a$, the regional boundary curve $C_{S_{1}^{\prime },D^{\prime \prime }}$ cannot be written as a unique functional expression, as schematically shown in Figure 4c. It is one solution to adjust the angle of the coordinate system to facilitate the integration expression. For example, for Figure 4c, set the direction of the line, where nodes $s_{1}$ and $s_{3}$ are located as the horizontal or vertical axis of the coordinate system, and then choose the appropriate order of integration. Moreover, if the distance between nodes does not meet the conditions for the existence of boundary curves, the information map is relatively simplified, as exemplified in Figure 4d when $d_{s_{1},s_{3}}<\frac{\lambda_{t}}{\lambda_{d}}\cdot 2\Delta t$ and $d_{s_{2},s_{3}}<\frac{\lambda_{t}}{\lambda_{d}}\cdot 2\Delta t$.

Theoretically, when nodes $s_{2}$ and $s_{3}$ are very close to each other, activating node $s_{3}$ after activating node $s_{2}$ can obtain very little incremental information due to the strong spatial correlation. In other words, it may not be a good choice to activate nodes alternately at this time. That is, it is better to activate node $s_{1}$ after activating node $s_{2}$ at this time, and the scheduling order of the nodes is 1213⋯. The boundary situation of the above two scheduling situations meets the condition as $info_{\left[2,1,3\right]}\left(s_{3}\right)=info_{\left[2,1,3\right]}\left(s_{1}\right)$. However, it is challenging to seek the exact analytical solution, and only the approximate numerical solution can be obtained by a numerical calculation. The specific solution algorithm can use the dichotomy method, and the specific calculation results will be given in Section 5.

The remaining possible scheduling cases, and the boundary conditions between different scheduling cases, are mainly shown in Table 2. Due to the limitation of space, other cases are not explained in detail here, and the general analysis idea is similar to the previous one; interested readers can refer to the arxiv version of this paper [32].

### 3.2. Three-Node General Triangular Layout

When three nodes present a general triangular layout, at least three variables are required to describe a general triangle. Due to the unintuitiveness of the three-dimensional diagram and the difficulty of theoretical analysis, the specific operation in this subsection is to keep the positions of the two nodes with the largest distance unchanged, traversing the different positions of the other node and analyzing the possible scheduling results. For detail, it is assumed that the distance *d* between nodes $s_{2}$ and $s_{3}$ is the largest, and they are located at (0,0) and (d,0), respectively, in the coordinate system. The traversed area can be reduced by half according to the horizontal symmetry. Then node $s_{1}$ needs to traverse the area as illustrated in the Figure 3b, where the shaded area is the optional position of node $s_{1}$.

When the position of node $s_{1}$ is selected on the boundary of the traversal region, the three nodes present an isosceles triangle. According to the previous analysis, the possible scheduling situation can be roughly obtained. For example, as the vertical height of node $s_{1}$ increases on the perpendicular bisector of nodes $s_{2}$ and $s_{3}$, the final activation proportion of node $s_{1}$ may gradually increases from 0 to $\frac{1}{3}$. In addition, if traversing from the top to the lower left on the arc boundary, the scheduling situation may change from an alternating activation to a situation where the node $s_{3}$ activation accounts for a half, i.e., scheduling sequence 3132⋯. Based on the above analysis, the possible scheduling results under the general triangular layout can be obtained, as shown in Table 3.

Therefore, the node positions can be traversed in the positive direction from the horizontal axis, and the vertical values can be traversed sequentially in the case of fixed values of each horizontal axis; then, the numerical solutions of the critical case equations in Table 3 can be found, respectively, to determine the critical layout between the different scheduling results.

## 4. Long-Term Optimal Mechanism

The single-step mechanism only concerns the current gain for each decision, and it does not consider the possible impact of the current decision on the future. The main scheme adopted in this section is to model the process as a Markov decision process, taking the current information residual map of the system as the state, and the number of system states is finite. Then, the Q-learning algorithm can be used to converge to the optimal scheduling result after a finite number of training times.

The main scheme adopted in this section is to model the process as a Markov decision process, taking the current information residual map of the system as the state. The information gain obtained by the node activated at the current moment is only related to the current information residual map, independent of the residual information map at all previous moments. Since the currently analyzed system contains three nodes and activates a node periodically, the number of system states is finite. Then, the Q-learning algorithm can be used to eventually converge to the optimal scheduling result after a finite number of training steps.

### 4.1. States, Actions, and Rewards

The state-space State mainly records the current information residual map of the system and can directly take the AoI of the latest sensed data of each node, as follows:

(42) $$State=AoI=\left[AoI\left(s_{1}\right),AoI\left(s_{2}\right),AoI\left(s_{3}\right)\right].$$

Since the decision time interval is fixed as Δt, the AoI can be abbreviated only as its coefficient about Δt. From the node spatio-temporal correlation and the previous analysis, it is known that the system takes the same action continuously with poor information gain. Thus, it can be stipulated by default that the system will not take the same action twice and more consecutively, which can reduce the total number of states and improve the training efficiency.

The survival time of data information of each node is limited for the reason that it will always be covered by new data of adjacent nodes after a certain time. Thus, the AoI of each node data has a maximum, greater than which the data contain no valuable information, and the AoI can be abbreviated as inf. The maximum AoI of each node can be calculated as Equation (44), where

(43) $$K_{s_{i},s_{j}}=\left\lfloor \frac{d_{s_{i},s_{j}}}{\frac{\lambda_{t}}{\lambda_{d}}\cdot \Delta t}\right\rfloor ,$$

and i,j,k∈{1,2,3}, i≠j≠k. In addition, $s_{j}$ and $s_{k}$ represent the two nodes whose data AoI takes the values of 1 and 2, respectively.

(44) $$\begin{array}{c} AoI{\left(s_{i}\right)}_{\max}=\max\left\{\min\left(K_{s_{i},s_{j}}+AoI\left(s_{j}\right),K_{s_{i},s_{k}}+AoI\left(s_{k}\right)\right)|AoI\left(s_{j}\right),AoI\left(s_{k}\right)\in \left\{1,2\right\},\right\} \end{array}$$

The total number of states of the system is shown in Equation (45),

(45) $$\begin{array}{cc} N_{State}=\sum_{i=1}^{3}N_{State}{|}_{AoI_{max}=AoI{\left(s_{i}\right)}_{max}}=\sum_{i=1}^{3}\sum_{m=1}^{AoI{\left(s_{i}\right)}_{max}}N_{State}{|}_{AoI_{max}=AoI\left(s_{i}\right)=m} & \\ =\sum_{i=1}^{3}\{1+\sum_{m=2}^{AoI{\left(s_{i}\right)}_{max}}[u\left(K_{s_{i},s_{j}}-\left(m-1\right)\right)\cdot u\left(K_{s_{i},s_{k}}-\left(m-2\right)\right)+ & \\ u\left(K_{s_{i},s_{j}}-\left(m-2\right)\right)\cdot u\left(K_{s_{i},s_{k}}-\left(m-1\right)\right)]\} \end{array}$$

where u(.) is a step function, as follows:

(46) $$u\left(x\right)=\left\{\begin{array}{c} 1\;x\geq 0\\ 0\;x<0 \end{array}\right.$$

The set of actions is $\left\{s_{1},s_{2},s_{3}\right\}$, where $s_{1}$, $s_{2}$ and $s_{3}$ represent the activation of the corresponding nodes, respectively. The state transfer process is as follows. Firstly, the system performs an action each time and activates the corresponding node, and the AoI value of the corresponding node is set to 0. Secondly, the AoI value of all node data plus 1 elapsed time is Δt.

Then, determine whether the AoI value of the node data exceeds the maximum value, and if it exceeds the maximum value, it is recorded as inf.

The immediate reward for each action is the incremental amount of information acquired, that is

(47) $$Reward=\frac{I_{gain}\left(State,s_{i}\right)}{I_{gain}\left(\left[inf,\;inf,\;inf\right],s_{1}\right)},\;i\in \left\{1,2,3\right\},$$

where the denominator is the information increment that the system can obtain by activating the node for the first time, whose role is to normalize the reward.

### 4.2. Q Learning Algorithm

The training process mainly adopts a greedy strategy, which means that the agent mainly takes random actions to explore the environment in the initial stage, and gradually increases the greedy coefficient as the number of training steps increases, i.e., the agent tends to choose the action with a larger Q value. The optimal long-term scheduling is obtained by waiting for the almost complete convergence of the Q-table. The process of updating the Q value for each training is as follows [33]

(48) $$Q_{new}\left(s,a\right)\leftarrow Q\left(s,a\right)+\alpha \left(R\left(s^{\prime }\right)+\gamma \max_{a^{\prime }}Q\left(s^{\prime },a^{\prime }\right)-Q\left(s,a\right)\right),$$

where *s* denotes the current state, *a* denotes the action, and Q(s,a) is the previous Q value. Meanwhile, α is the learning rate, and γ is the discount factor. The *R* is the immediate reward observed in the new state $s^{\prime }$, and the $max_{a^{\prime }}Q\left(s^{\prime },a^{\prime }\right)$ represents the estimate of optimal future reward from the next state $s^{\prime }$.

## 5. Numerical and Simulation Results

### 5.1. Scheduling Results with Single-Step Optimal Mechanism

Without a loss of generality, the value of Δt is taken as 1 in this section.

#### 5.1.1. Three-Node Isosceles Triangle Layout

**Figure 5:**
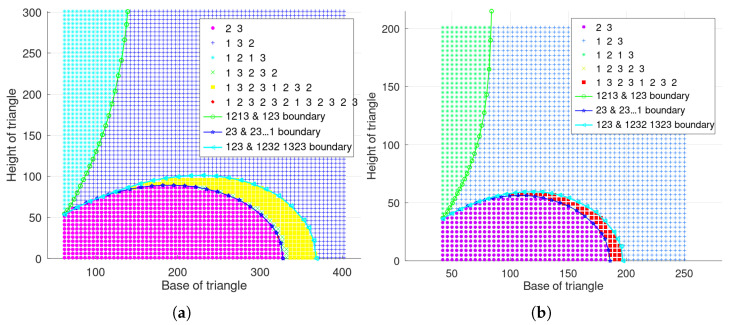
Scheduling results of single step mechanism with three nodes presenting an isosceles triangle. (**a**) $\lambda_{d}=0.01$, $\lambda_{t}=0.3$. (**b**) $\lambda_{d}=0.025$, $\lambda_{t}=0.5$.

When the traversal step is 5 with parameters $\lambda_{d}=0.01$ and $\lambda_{t}=0.3$, the main scheduling results are illustrated in Figure 5a, where the horizontal axis represents the values of the base of the isosceles triangle, and the vertical axis represents the height of the isosceles triangle. The scatter points of different shapes in Figure 5a represent a different type of scheduling situation, and their corresponding periodic scheduling sequences are shown in the legend of the figure, respectively. The three curves are the approximate numerical bounds on the critical scheduling case obtained from the previous theoretical analysis and numerical calculation. Specifically, the curve marked by circles represents the boundary node distribution for the cycle scheduling sequence of 1213 and 123. The curve marked by triangles represents the boundary node distribution for the cycle scheduling sequence of 123 and the scheduling result of 12321323⋯. In addition, the curve marked with pentagrams represents the critical distribution of the scheduling sequence 23 and 23⋯1, and the interval between two node $s_{1}$ activations is not greater than $\left\lceil \frac{\lambda_{d}\cdot d_{s_{1},s_{2}}}{\lambda_{t}\cdot \Delta t}\right\rceil =\left\lceil \frac{\lambda_{d}\cdot \sqrt{h_{th}^{2}+d_{th}^{2}/4}}{\lambda_{t}\cdot \Delta t}\right\rceil$, where $h_{th}$ and $d_{th}$ correspond to the values taken on the boundary curve. In addition, when the traversal step is 4 with $\lambda_{d}=0.025$ and $\lambda_{t}=0.5$, the result is shown in the Figure 5b.

#### 5.1.2. Three-Node General Triangular Layout

**Figure 6:**
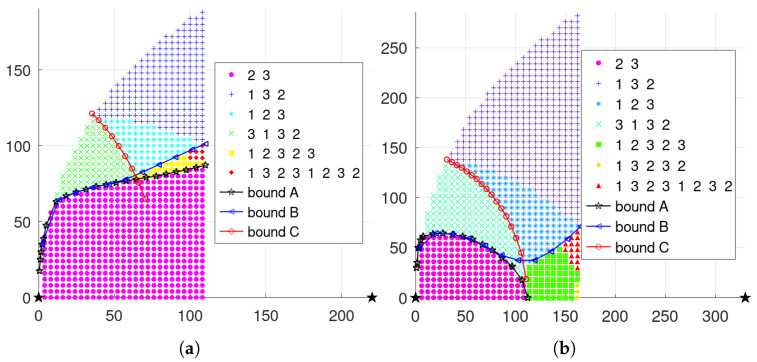
Single-step mechanism results with general triangle layout when $\lambda_{d}=0.01$ and $\lambda_{t}=0.3$. (**a**) $d_{s_{2},\;s_{3}}=220$. (**b**) $d_{s_{2},\;s_{3}}=330$.

When $\lambda_{d}=0.01$ and $\lambda_{t}=0.3$, the scheduling results with the maximum distance between nodes $d_{s_{2},\;s_{3}}=280$ and $d_{s_{2},\;s_{3}}=450$ are, respectively, illustrated in Figure 6a,b, where the three curves correspond to the boundary conditions in Table 3.

### 5.2. Scheduling Results with Long-Term Optimal Mechanism

**Figure 7:**
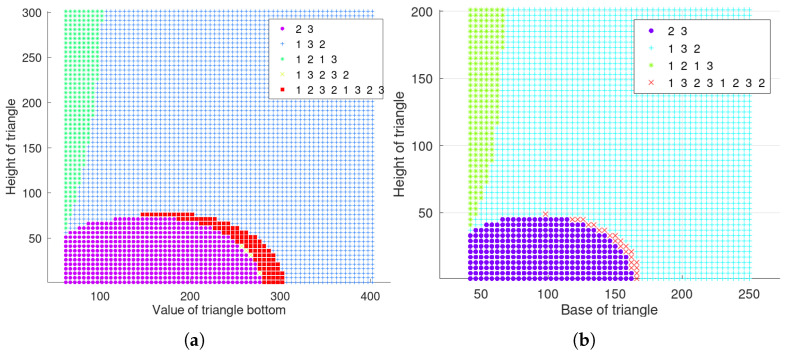
Scheduling results of long-term mechanism with three nodes presenting an isosceles triangle. (**a**) $\lambda_{d}=0.01$, $\lambda_{t}=0.3$. (**b**) $\lambda_{d}=0.025$, $\lambda_{t}=0.5$.

To facilitate comparison with the single-step mechanism results, some parameter values are the same. In addition, the learning rate α is 0.1, and the discount factor γ is 0.9. When three nodes present an isosceles triangular layout with parameters $\lambda_{d}=0.01$ and $\lambda_{t}=0.3$, the final obtained scheduling results are shown in Figure 7a, where the optimal cycle scheduling sequence for each layout is shown in the legend. Moreover, the scheduling results are shown in Figure 7b with parameters $\lambda_{d}=0.025$, $\lambda_{t}=0.5$.

**Figure 8:**
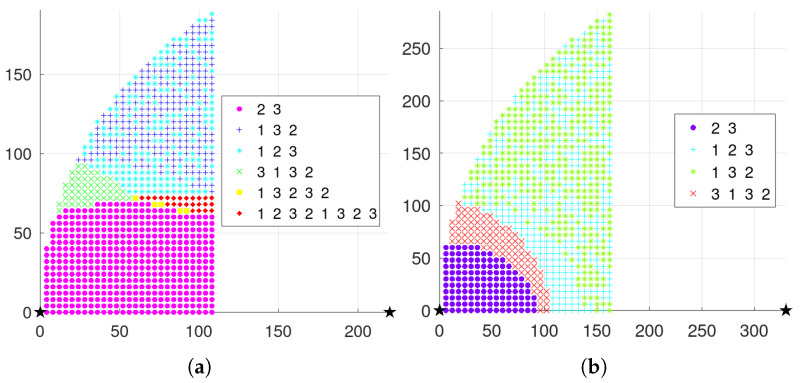
Long-term mechanism results with general triangle layout when $\lambda_{d}=0.01$ and $\lambda_{t}=0.3$. (**a**) $d_{s_{2},\;s_{3}}=220$. (**b**) $d_{s_{2},\;s_{3}}=330$.

Meanwhile, the scheduling results are shown in Figure 8a when three nodes present a general triangular layout with $\lambda_{d}=0.01$, $\lambda_{t}=0.3$, and $d_{s_{2},\;s_{3}}=280$. In addition, the the scheduling results are shown in Figure 8b with $ds_{2},\;s_{3}=450$.

### 5.3. Performance Comparison of Two Mechanisms

The scheduling results with the single-step mechanism and the long-term mechanism are the same in quite a few regions, which can be observed specifically in combination with Figure 5 and Figure 7, or Figure 6 and Figure 8. However, there are some regions where the results of the two mechanisms are not the same; that is, the results of the single-step mechanism in these regions are not the results meeting the long-term mean optimum. Therefore, in the long-term, it is necessary to select a specific moment to select the second-best decision, which may be able to bring more future benefits. Meanwhile, the fluctuation of the incremental information obtained under the long-term mechanism should be slightly larger.

When the three nodes show an isosceles triangular layout and the traversal step is 5 with $\lambda_{d}=0.01$, $\lambda_{t}=0.3$, the highest mean information increment with a long-term mechanism is about 2.5% higher than that with a single-step mechanism. Regarding the node layouts with different results obtained by two mechanisms, the mean information increment with a long-term mechanism is about 0.8% higher on average than the other one. As for all layout cases, the mean information increment with the long-term mechanism is slightly higher by about 0.1% on average. The standard deviation of the incremental information acquisition with the long-term mechanism is also larger, and it is on average about 60% higher in the situation of node layouts for which the two mechanisms obtain different results. Similarly, when the three nodes are distributed in a general triangle and traversal step length is 4 with $d_{s_{2},\;s_{3}}=220,\lambda_{d}=0.01$, and $\lambda_{t}=0.3$, the mean information increment obtained by the long-term mechanism is up to 2.1% higher than that of the single-step mechanism, which is about 0.6% higher on average for the situation of node layouts with different results obtained by the two mechanisms. As for all layout cases, the mean information increment with the long-term mechanism is slightly higher by about 0.1% on average. On the other hand, the standard deviation with the long-term mechanism is about 80% higher on average in the node layout cases with different scheduling results for the two mechanisms. In summary, the mean information increment with long-term mechanism is slightly larger, but the standard deviation is also slightly larger.

### 5.4. Complexity Analysis

The computation of both mechanisms can be conducted offline when the correlation coefficient parameters are determined. The time complexity of the offline computation of the single-step mechanism is O(N) at most, where *N* is the number of all valid states of AoI. If the system requires obtaining decision actions for all AoI states with the single-step mechanism, the offline computation complexity would be O(N), and the computation speed largely depends on the computation speed of the numerical integration. The program can utilize a memory storage pool to record encountered states and selected actions for subsequent queries, keeping the space complexity within O(N). If the system only focuses on the periodic scheduling results with the single-step mechanism, the time complexity would be O(m), where *m* represents the corresponding period of the scheduling result.

On the other hand, the long-term mechanism requires a Q-table to record the values of corresponding actions for all states, resulting in a space complexity of O(N). The time complexity is not directly measurable, as the algorithm requires training for multiple rounds until the Q-table converges. However, overall, the computation speed is slower compared to the single-step mechanism. When the correlation coefficients remain unchanged, the system can directly obtain scheduling results offline. Therefore, if the system is used in actual online applications, there is no need for additional computational costs. However, if adjustments to the correlation coefficient parameters are necessary, recalculations are required, and the majority of computational consumption could be attributed to the calculation of numerical integration. This also reflects the limitation of the model algorithm when the correlation properties of the source are time-varying. Therefore, considering the performance and resource consumption, in practical deployment applications, the single-step decision mechanism may have a slight advantage.

## 6. Experimental Evaluation

In this section, the relative humidity grid dataset [34,35,36,37,38] is used to evaluate our proposed scheduling mechanism. In the first part, the data are analyzed to extract the scaling parameters of the covariance model described earlier. In the second part, four scheduling methods are compared in terms of their two-dimensional sense performance. In the third part, a summary and discussion are presented, illustrating the model’s performance and limitations.

### 6.1. Data Analysis

In order to fully reflect the stochastic variation among the data, a down-sampling operation with a step size of two is performed on the grid data; then, a circular grid area data with a radius of 15 is selected to compare the global sensing performance of different scheduling methods.

The Pearson correlation coefficient formula is used to calculate the spatio-temporal correlation of the grid data. Firstly, the correlation coefficients about the spatial distance between the same moments of grid data are calculated, and then the $\lambda_{d}$ can be fitted. Secondly, the time correlation between different moments of the same grid data is calculated, and the $\lambda_{t}$ can be fitted. The joint spatio-temporal correlation between the data is then verified by calculating the correlation coefficient when both the distance and time difference between different grid data are not zero. If the fitting results deviate from the actual results, it may affect the performance of the subsequent scheduling.

### 6.2. Performance Comparison

The specific implementation steps of the experiment are as follows: within the circular region mesh data, an equilateral triangle of suitable size is selected, and two of the vertices are set as the location of the node, while the traversal region of the other node remains as shown in Figure 3b.

The metric of the experiment is the mean square error over the entire area. The effective coverage area of each node data can be determined by the previous analysis. Within the effective coverage area of each node, the corresponding conditional distribution mean based on the correlation coefficient between each location and the node position is used as the estimated data for that location. The scheduling methods include the single-step and long-term mechanisms previously proposed in this paper, and the other two are the ideal scheduling and STI-based scheduling, respectively. The ideal scheduling method is that the system has all the grid data and makes the scheduling decision that provides the minimum total scope error.

Currently, there is relatively limited research on effective information for modeling the joints of nodes in the spatio-temporal domain. Most of the existing literature focuses on the effective information at individual node locations. Therefore, as an comparison approach abbreviated as STI-based scheduling, we referred to reference [30], which uses point spatially and temporally correlative mutual information at the sensor location as a metric for system scheduling. In other words, in the modeling scenario of this paper, the STI-based scheduling activates the node with the least point of spatially and temporally information based on the point of spatially temporally information of each node location at each moment.

This method is similar to the approach in this paper. However, the main difference between the two methods proposed in this paper and the referenced method is that the former integrates the node information over the entire spatio-temporal domain and uses it as a measure for information quantification.

**Figure 9:**
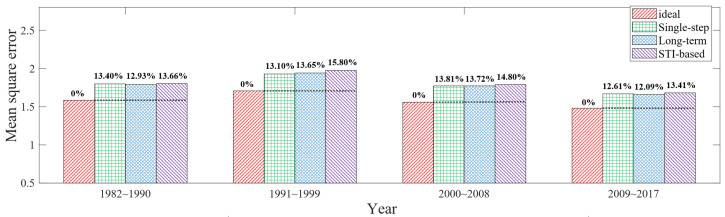
Experimental results of global circular grid data.

The time span of each evaluation is nine years, which are 1982–1990, 1991–1999, 2000–2008, 2009–2017, respectively. Moreover, the model parameters were extracted mainly using the data from the first three years used in each experiment. The experimental results are shown in Figure 9. The ideal scheduling method has the best performance with the minimum scope mean square error.

The STI-based method has the maximum mean square error, averaging 14.42% higher than the ideal scheduling method. The performance of single-step and long-term mechanisms lies between the two aforementioned methods, with the mean square error that is on average 13.16% higher than that of the ideal scheduling method. Since two mechanisms obtain the same scheduling results in many node layouts, and the global average difference in the information mean obtained by two mechanisms is less than 1% in simulation, the global performance difference caused by two methods in the experiment is also minimal, as shown in Figure 9.

In addition, due to the higher variance in information acquisition of the long-term mechanism compared to the single-step mechanism, and considering the impact of error and randomness in data parameter fitting, it is possible that the long-term mechanism may exhibit slightly inferior results compared to the single-step mechanism in experiments, as shown in Figure 9. Furthermore, since the scheduling results of the two mechanisms are the same in many node layout scenarios, the average performance difference between them is very small, with minor fluctuations present.

**Figure 10:**
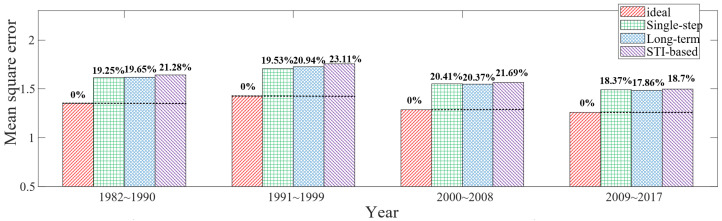
Experimental results of adaptive regional grid data.

If the reasonableness of node layout within the information sense region is considered when traversing node locations, it is not very reasonable to uniformly examine the total scope error in the circular region. For example, when two nodes are very close to each other, the effective coverage of three nodes may converge to an ellipse. For this consideration, circles of appropriate size are selected with each node position in the experiments; then, the joint coverage of the three circles is set as the data evaluation scope for examining the model performance. Under the above experimental operation, the performance comparison of the four scheduling methods is obtained, as shown in Figure 10. The single-step and long-term mechanisms have the mean square error that is on average 19.54% higher than that of the ideal scheduling method. The mean square error of the STI-based scheduling method is on average 21.20% higher than that of the ideal scheduling method.

### 6.3. Summary and Discussion

The above experimental results demonstrate that considering the spatio-temporal scope effective information of sensor nodes, as opposed to solely considering the point information at their individual positions, can guide system decisions and improve perception accuracy in area monitoring applications of the IoT. Therefore, in resource-constrained IoT deployment scenarios in practical real-world settings, the use of a spatio-temporal information model provides an optimized method for efficient node activation decisions, thereby improving the overall system sensing with accuracy and energy efficiency. This will potentially provide further support for the development of efficient perception in the future of the IoT.

It is known that the correctness of the correlation parameter fit is the primary factor in determining the model performance. In addition, whether the adopted covariance function model fits the distribution of the data set is also an important factor. In the case of stochastic processes, the correlation model may not be stationary, i.e., the correlation coefficients may be time-varying. The limitations of the current model mainly come from the above aspects, which may be addressed in future studies.

Furthermore, sensing nodes in IoT applications may experience changes in their positions due to intrinsic or extrinsic factors, making their locations non-static. This implies that the mobility of nodes is also worth considering and studying. In other words, node mobility can be broadly classified into active and passive mobility. In the case of passive mobility, sensor nodes may be influenced by external environmental factors, leading to random movements in their positions. In such cases, it may be necessary to predict the passive motion trajectories of multiple nodes, estimate their distributions, and adjust system decisions accordingly. On the other hand, an example of a typical application scenario involving the active mobility of nodes is the current stage of Unmanned Aerial Vehicle (UAV) surveillance. When nodes possess the ability to actively move, a preliminary consideration is to establish a metric that measures the trade-off between the energy consumption for node movement and the amount of effective information acquired. This metric aims to quantify the information gain from mobile data collection. By integrating the planning of multiple node trajectories, it is possible to investigate optimization decision algorithms for maximizing system energy efficiency. These aspects require further modeling and analysis and are important research directions for the future work on this paper.

## 7. Conclusions

In this paper, a SSIM is developed to quantify the valuable information of sensor data, which decays with space and time. Two optimal scheduling mechanisms are proposed. One is the single-step mechanism, and the approximate numerical bounds for node layout between partial scheduling results are obtained by theoretical analysis and numerical calculation, which coincide with the simulation results. The other one is the long-term mechanism, which is modeled as a Markov decision process, and the optimal scheduling results with different node layouts are obtained using the Q-learning algorithm. Through simulation and experiments, the scheduling results of both mechanisms are the same in many node layout cases. In few node layouts, the mean incremental information obtained by the long-term mechanism is up to about 2% higher than that of the single-step mechanism, but the standard deviation with the long-term mechanism is also higher than that of the single-step mechanism. The average performance of the two mechanisms is similar under all node layouts, and the long-term mechanism is slightly higher. In future work, we may focus on the SSIM with time-varying covariance functions and node mobility, and study the cooperative scheduling of multiple nodes to improve energy efficiency and extend lifetime.

## Figures and Tables

**Table 1:** Comparison of References.

References	Information Source	Sensors	Metrics	Research Results
[18]	Single-point	Two	Estimation Error	Optimal time shift between two sensors
[19,20]	Multi-point	Multiple	Estimation Error	DRL-based scheduling mechanism
[22,23]	Single-point (Noisy Ornstein-Uhlenbeck process)	Single	Mutual information	Closed-form VoI expressions
[24,25]	Regional	Multiple	Error-tolerable sensing (ETS) coverage	AoI violation probability, Optimal sensors’ transmission power
[26,27]	Time-varying Gauss–Markov Random Field (GMRF)	Multiple	Mean squared Estimation error	Closed-form expressions for estimation error, optimal spatial-temporal Sampling rates
[28,29]	N Gaussian process	Multiple	Mean squared error (MSE)	Optimal scheduling policy
[30]	Single-point	Multiple	Spatial-temporal mutual information	Optimal update interval
[31]	Multi-point	Multiple	Overall utility of information update	Status update node set
This work	Regional	Three	Spatial-temporal Scope information	Node activation strategy in any layout

**Table 2:** Single-step mechanism scheduling situations when three nodes present an isosceles triangle.

Typical Layout	Scheduling Results	Conditions
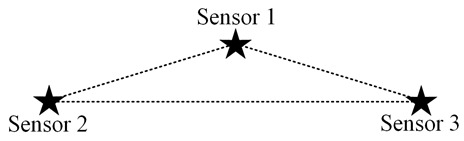	2323⋯	$info_{\left[\inf,\;2,\;1\right]}\left(s_{2}\right)>info_{\left[\inf,2,1\right]}\left(s_{1}\right)$
	Boundary	$info_{\left[\inf,2,1\right]}\left(s_{2}\right)=info_{\left[\inf,2,1\right]}\left(s_{1}\right)$
	⋮	⋮
	12321323⋯	$info_{\left[3,2,1\right]}\left(s_{1}\right)<info_{\left[3,2,1\right]}\left(s_{2}\right)$ $info_{\left[2,1,3\right]}\left(s_{3}\right)>info_{\left[2,1,3\right]}\left(s_{1}\right)$ $info_{\left[1,3,2\right]}\left(s_{2}\right)>info_{\left[1,3,2\right]}\left(s_{3}\right)$ $info_{\left[4,2,1\right]}\left(s_{1}\right)>info_{\left[4,2,1\right]}\left(s_{2}\right)$
	Boundary	$info_{\left[3,2,1\right]}\left(s_{1}\right)=info_{\left[3,2,1\right]}\left(s_{2}\right)$
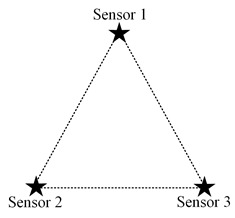	123123⋯	$info_{\left[3,2,1\right]}\left(s_{1}\right)>info_{\left[3,2,1\right]}\left(s_{2}\right)$ $info_{\left[2,1,3\right]}\left(s_{3}\right)>info_{\left[2,1,3\right]}\left(s_{1}\right)$ $info_{\left[1,3,2\right]}\left(s_{2}\right)>info_{\left[1,3,2\right]}\left(s_{3}\right)$
	Boundary	$info_{\left[2,1,3\right]}\left(s_{3}\right)=info_{\left[2,1,3\right]}\left(s_{1}\right)$
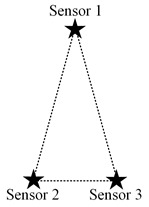	12131213⋯	$info_{\left[2,1,3\right]}\left(s_{3}\right)<info{}_{\left[2,1,3\right]}\left(s_{1}\right)$ $info_{\left[1,2,4\right]}\left(s_{3}\right)>info{}_{\left[1,2,4\right]}\left(s_{2}\right)$ $info_{\left[1,4,2\right]}\left(s_{2}\right)>info{}_{\left[1,4,2\right]}\left(s_{3}\right)$ $info_{\left[2,3,1\right]}\left(s_{2}\right)<info{}_{\left[2,3,1\right]}\left(s_{1}\right)$

**Table 3:** Single-step mechanism scheduling situations when three nodes present an general triangle.

Typical Layout	Scheduling Results	Conditions
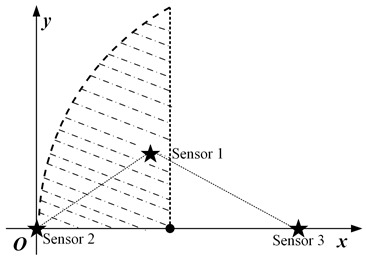	2323⋯	$info_{\left[\inf,2,1\right]}\left(s_{2}\right)>info_{\left[\inf,2,1\right]}\left(s_{1}\right)$
	Boundary A	$info_{\left[\inf,2,1\right]}\left(s_{2}\right)=info_{\left[\inf,2,1\right]}\left(s_{1}\right)$
	⋮	⋮
	12321323⋯	$info_{\left[3,2,1\right]}\left(s_{1}\right)<info_{\left[3,2,1\right]}\left(s_{2}\right)$ $info_{\left[2,1,3\right]}\left(s_{3}\right)>info_{\left[2,1,3\right]}\left(s_{1}\right)$ $info_{\left[1,3,2\right]}\left(s_{2}\right)>info_{\left[1,3,2\right]}\left(s_{3}\right)$ $info_{\left[4,2,1\right]}\left(s_{1}\right)>info_{\left[4,2,1\right]}\left(s_{2}\right)$
	Boundary B	$info_{\left[3,2,1\right]}\left(s_{1}\right)=info_{\left[3,2,1\right]}\left(s_{2}\right)$
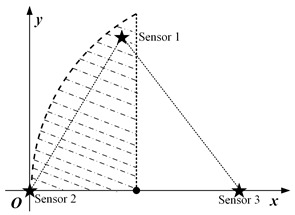	123123⋯	$info_{\left[3,2,1\right]}\left(s_{1}\right)>info_{\left[3,2,1\right]}\left(s_{2}\right)$ $info_{\left[2,1,3\right]}\left(s_{3}\right)>info_{\left[2,1,3\right]}\left(s_{1}\right)$ $info_{\left[1,3,2\right]}\left(s_{2}\right)>info_{\left[1,3,2\right]}\left(s_{3}\right)$
	Boundary C	$info_{\left[1,3,2\right]}\left(s_{3}\right)=info_{\left[1,3,2\right]}\left(s_{2}\right)$
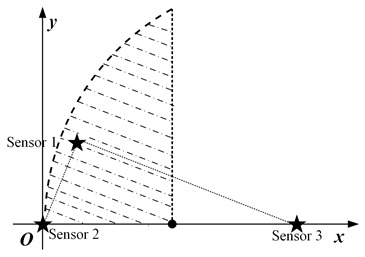	31323132⋯	$info_{\left[2,4,1\right]}\left(s_{2}\right)>info_{\left[2,4,1\right]}\left(s_{1}\right)$ $info_{\left[1,3,2\right]}\left(s_{3}\right)>info_{\left[1,3,2\right]}\left(s_{2}\right)$ $info_{\left[4,2,1\right]}\left(s_{1}\right)>info_{\left[4,2,1\right]}\left(s_{2}\right)$ $info_{\left[3,1,2\right]}\left(s_{3}\right)<info{}_{\left[3,1,2\right]}\left(s_{1}\right)$

## Data Availability

Not applicable.
